# Influencing factors and clinical significance of the metastatic lymph nodes ratio in gastric adenocarcinoma

**DOI:** 10.1186/1756-9966-28-55

**Published:** 2009-04-26

**Authors:** Ji-wei Yu, Ju-gang Wu, Lin-hai Zheng, Biao Zhang, Xiao-chun Ni, Xiao-qiang Li, Bo-jian Jiang

**Affiliations:** 1 Department of General Surgery, No 3 People's Hospital, Shanghai Jiao-Tong University School of Medicine Shanghai 201900, PR China; 2 Department of Pathology, No 3 People's Hospital, Shanghai Jiao-Tong University School of Medicine Shanghai 201900, PR China

## Abstract

**Background:**

To investigate influencing factors of the metastatic lymph nodes ratio (MLR) and whether it is related to survival in patients with gastric adenocarcinoma.

**Methods:**

We retrospectively evaluated the clinical features of 121 patients with gastric adenocarcinoma enrolled in our hospital between 2000 and 2007. The receiver operating characteristic (ROC) curve was used to determine the cutoff of the MLR, and CK20 immunohistochemical staining was used to detect micrometastasis of the lymph nodes.

**Results:**

The areas under the ROC curve of MLR used to predict the death of 3-year and 5-year postoperative patients were 0.826 ± 0.053 and 0.896 ± 0.046. Thus MLR = 30.95% and MLR = 3.15% were designated as cutoffs. The MLR was then classified into three groups: MLR_1 _(MLR<3.15%); MLR_2_(3.15% ≤ MLR ≤ 30.95%); and MLR_3 _(MLR>30.95%). We found that patients with a higher MLR demonstrated a much poorer survival period after radical operation than those patients with a lower MLR (P = 0.000). The COX model showed that MLR was an independent prognostic factor (P = 0.000). The MLR could also discriminate between subsets of patients with different 5-year survival periods within the same N stage (P < 0.05). The MLR has been shown to be 34.7% (242/697) by HE staining and 43.5% (303/697) by CK staining (P = 0.001). The clinicopathological characteristics of lymph vessel invasion and the depth of invasion could significantly affect the MLR.

**Conclusion:**

MLR is an independent prognostic factor in gastric cancer. The combined ROC curve with MLR is an effective strategy to produce a curve to predict the 3-year and 5-year survival rates.

## Background

The metastatic lymph nodes ratio (MLR, N ratio) is a powerful independent prognostic factor in gastric cancer, even when only a few lymph nodes metastases were found [1-6]. The MLR reflects the efficacy of the resection of lymph nodes, which is the best method to prevent stage migration [3,4]. However, the criteria for MLR classification are controversial. In order to investigate the relationship between MLR and prognosis, N stage, and clinical characteristics, we used a receiver operating characteristic curve (ROC curve) to determine the MLR cutoff. Additionally, the influence of MLR on micrometastasis was also evaluated.

## Methods

### Patients

Between 2000 and 2007, 121 patients with gastric adenocarcinoma were enrolled in this study from the Department of General Surgery, No. 3 People's Hospital, Shanghai Jiao-Tong University School of Medicine. All patients were underwent a curative gastrectomy and none of the patients received preoperative treatments. These patients consisted of 77 men and 44 women, ranging in age from 29 to 82, with a median age of 64. Total gastrectomy was performed in 9 patients, distal subtotal gastrectomy in 90 patients, and proximal subtotal gastrectomy in 22 patients. Additionally, 2 patients underwent D1 lymphadenectomy, 110 patients underwent D2 lymphadenectomy, and 9 patients underwent D3 lymphadenectomy. Postsurgery pathological examination showed 16 early adenocarcinomas, 4 fungating type adenocarcinomas, 16 ulcerative type adenocarcinomas, 71 invasion ulcerative type adenocarcinomas, and 14 diffuse infiltrative type adenocarcinomas. All clinicopathological profiles were evaluated in accordance with the criteria of the Japanese Gastric Cancer Association [7]. Moreover, N stage was also evaluated according to the TNM classification of the 6^th ^edition criteria of the International Union against Cancer (UICC) [8]. Patient follow-up ended on April 30, 2008 and the mean follow-up was 23 months. During the follow-up period, 46 patients died of recurrence or metastasis, 6 patients died of other diseases, and 20 patients were lost to follow-up. The survival time ranged from 6 to 93 months.

### Immunohistochemistry

CK20 immunohistochemical staining and hematoxylin-eosin (HE) staining were performed on 695 consecutive lymph node sections from 45 gastric cancer patients. The tissue sections were deparaffinized, dehydrated, and incubated in 3% hydrogen peroxide to block endogenous peroxidase activity. For the purpose of antigen retrieval, samples were microwaved for 10 minutes and were then washed with PBS. Immunohistochemical staining was performed with mouse monoclonal antibody against human CK20 primary antibodies (Changdao, Shanghai, China). Positive controls consisted of gastric cancer histological sections (Changdao, Shanghai, China), and negative controls used PBS in place of the primary antibody.

### Criterion of lymph node micrometastasis

CK20 is expressed in the cytoplasm. Lymph node sections with an N_0 _of HE staining, positive CK20 immunohistochemical staining, and a tumor diameter in the lymph nodes ranging from 0.2 to 2 mm were defined as lymph node micrometastasis. The results above were analyzed by two pathologists.

### Statistical analysis

All statistical calculations were performed using the SPSS 13.0 statistical software. ROC curves were used to assess the accuracy of the MLR prediction survival. Comparison of the MLR with CK20 immunohistochemical staining and HE staining was examined with a χ^2 ^test. Patient survival was analyzed using the Kaplan Meier product limit method. The log rank test was used to evaluate the difference between groups. The relationship between MLR and clinical characteristics was examined with the Mann-Whitney U test. Statistical significance was defined as P < 0.05.

## Results

### Postsurgery survival rate

Of all patients, the postsurgery 1-year to 7-year survival rates were 74%, 50%, 40%, 29%, 17%, 13%, and 8%, respectively.

### ROC curve analysis correlation between MLR and survival

**Figure 1:**
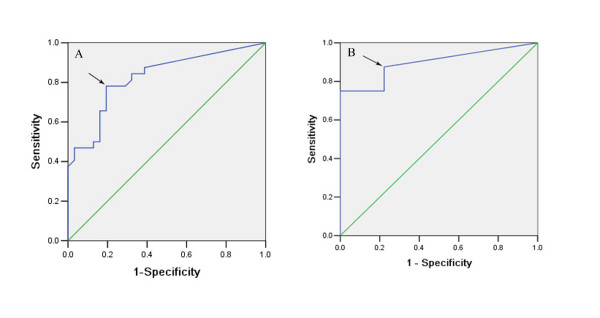
**ROC curve of MLR for predicting survival rate**. A. For predicting the 3-year survival rate; B. For predicting the 5-year survival rate.

After excluding from the original 121 patients that had died of other diseases or were lost to follow-up in 3 years, the ROC curve was drawn according to the survival of the remaining 63 patients (Figure 1A). Similarly, after excluding the patients that had died of other diseases or were lost to follow-up in 5 years, the ROC curve was drawn according to the survival of the remaining 49 patients (Figure 1B). The areas under the curves described above were 0.826 ± 0.053 (95% CI: 0.723 – 0.929) (*P *= 0.000) for the three-year survival ROC curve and 0.896 ± 0.046 (95% CI: 0.806 – 0.986) (*P *= 0.000) for the five-year survival curve. According to Youden's index, the maximum J value was 0.587 and 0.653, respectively (J = Sensitivity + Specificity - 1). Cutoffs of MLR = 30.95% (Figure 1A, arrow) and MLR = 3.15% (Figure 1B, arrow) were designated, respectively. Under these circumstances, the sensitivity was 78.1% and 87.5% and the specificity was 80.6% and 77.8%.

### Correlation between MLR grades and prognosis

**Figure 2:**
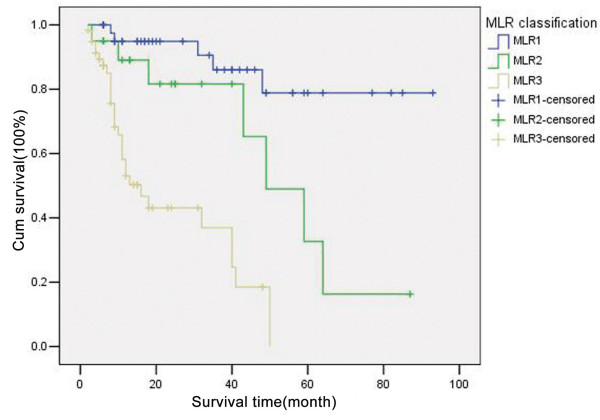
**Survival curves of patients in different MLR groups**.

With MLR = 30.95% and MLR = 3.15% designated as cutoffs, the MLR was defined as MLR_1 _(MLR<3.15%), MLR_2 _(3.15% ≤ MLR ≤ 30.95%), and MLR_3 _(MLR>30.95%). Univariate survival analysis suggested that a significant difference in prognosis was found among the different MLR groups (*X*^2 ^= 36.575, *P *= 0.000). Postsurgery survival time was shorter in patients with a higher MLR (Figure 2). As shown in Table 1, multivariate risk analysis showed that only MLR is an independent prognostic factor. Patients with a higher MLR suffered a higher death risk (RR = 2.801, P = 0.000, 95% CI: 1.680 – 4.668)(Table 2).

**Table 1:** Influence of clinicopathological characteristics on the prognosis in 121 gastric adenocarcinoma patients.

*Characteristics*	*Samples*	*Five-year survival (%)*	*Log-rank* *(X*^2^*value)*	*P value*
Gender (male/female)	77/44	35.5/49.5	0.527	0.468
Lauren type				
Intestinal type	109	46.1	6.322	0.012
Diffuse type	12	0		
Type of histology				
1–2	75	40.5	0.000	0.990
3	46	40.0		
Lymphatic vessel invasion				
Negative	54	60.6	14.199	0.000
Positive	67	18.3		
Blood vessel invasion				
Negative	100	43.7	13.455	0.000
Positive	21	28.8		
Lymph nodes metastasis				
Negative	44	79.0	24.919	0.000
Positive	77	13.0		
Depth of invasion				
T_1_	18	94.1	25.835	0.000
T_2_	31	56.0		
T_3_	31	36.7		
T_4_	41	0		
N stage (UICC)				
N_0_	43	78.9	34.320	0.000
N_1_	44	22.1		
N_2_	24	0		
N_3_	10	0		
N stage (JRSGC)				
N_0_	42	78.9	38.976	0.000
N_1_	38	12.6		
N_2_	31	16.4		
N_3_	10	0		
MLR				
MLR_1_	43	78.9	36.575	0.000
MLR_2_	20	32.7		
MLR_3_	58	0		

**Table 2:** Multivariate risk analysis of 121 gastric adenocarcinoma patients.

*Characteristics*	*B*	*S.E.*	*Wald*	*df*	*Sig.*	*Exp (B)*	*95.0%(CI))*
Lauren type	0.901	0.439	4.218	1	0.04	2.462	1.042 – 5.819
Depth of invasion	0.684	0.223	9.397	1	0.002	1.981	1.280 – 3.067
MLR	1.030	0.261	15.610	1	0.000	2.801	1.680 – 4.668

### Correlation between MLR and N stage in gastric adenocarcinoma

**Figure 3:**
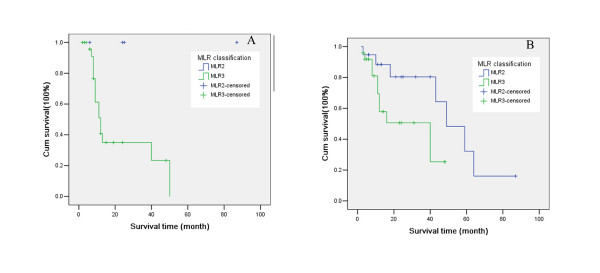
**Survival curves in patients with the same N stage, but in different MLR groups**. A. N_2 _stage (JRSGC classification); B. N_1 _(UICC classification).

As shown in Table 3, patients with the same N stage may be in different MLR groups. Moreover, in N_2 _stage (JRSGC classification), differences in the patients' prognosis were seen among the different MLR groups (*X*^2 ^= 4.372, *P *= 0.037) (Figure 3A). Similarly, in N_1 _stage (UICC classification), differences were also observed (*X*^2 ^= 4.320, *P *= 0.038) (Figure 3B).

**Table 3:** Correlation between MLR and N stage in gastric adenocarcinoma.

		*MLR groups [n (%)]*					*MLR groups [n (%)]*		
					
*N stage (UICC)*	*Samples*	MLR_1_	MLR_2_	MLR_3_	*N stage (JRSGC)*	*Samples*	MLR_1_	MLR_2_	MLR_3_
N_0_	43	43(100)			N_0_	43	43(100)		
N_1_	44		19(43.2)	25(56.8)	N_1_	38		16(42.1)	22(57.9)
N_2_	24		1(4.2)	23(95.8)	N_2_	30		4(13.3)	26(86.7)
N_3_	10			10(100)	N_3_	10			10(100)

### Effects of lymph node micrometastasis on the MLR in gastric adenocarcinoma

**Figure 4:**
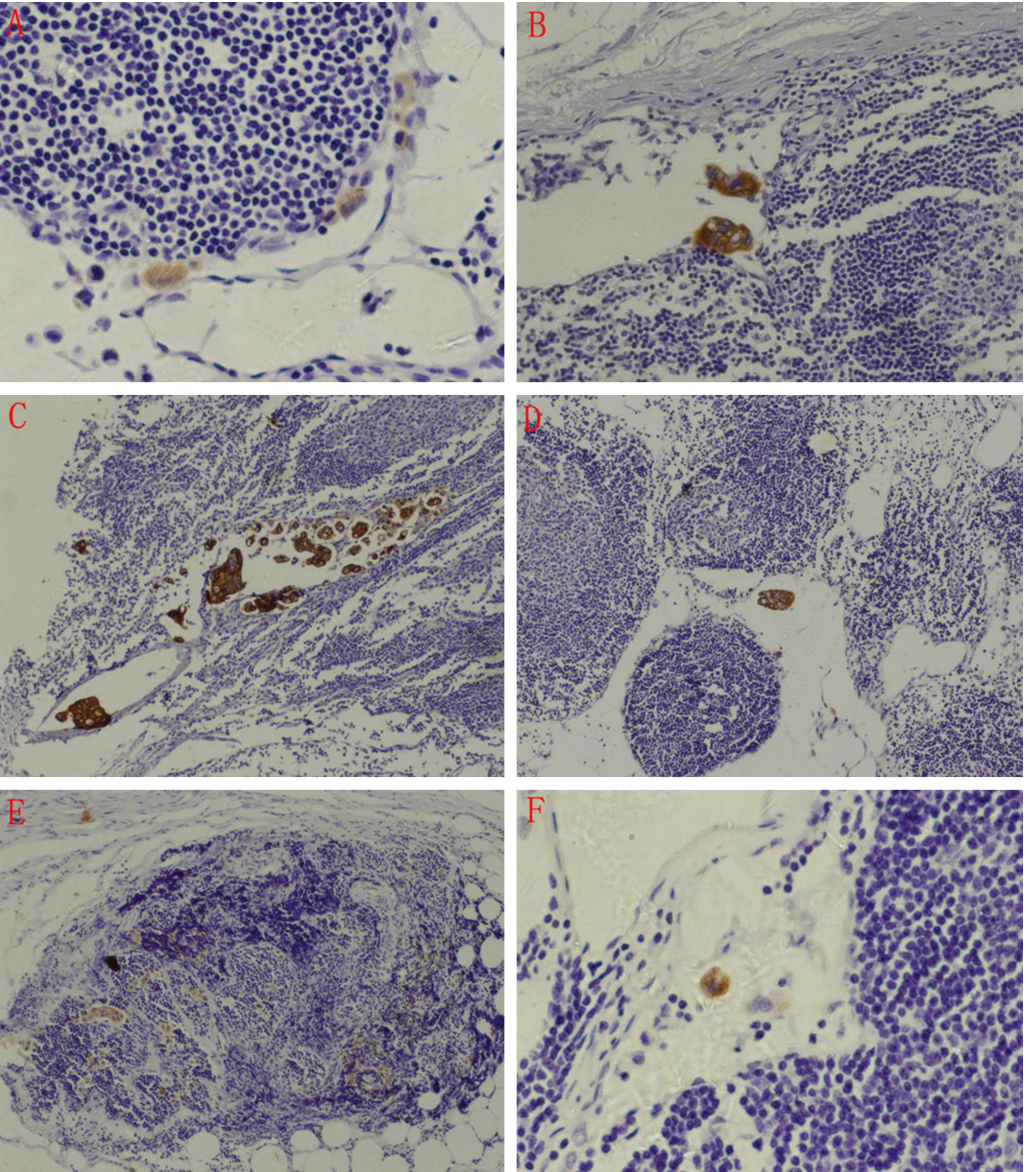
**Distribution characteristics of lymph node micrometastasis**. A. Marginal sinus type, nonclustered (×400); B. Marginal sinus type, clustered (×200); C. Intermediate sinus type, clustered and nonclustered (×100); D. Parenchymal type, clustered (×100); E. Diffuse type, clustered (×100); F. Isolated tumor cells (×400).

Lymph node micrometastasis was identified as a metastatic focus ranging from 0.2 to 2 mm in diameter and was mainly located at the marginal sinus with a nonclustered or clustered distribution. Occasionally, some were also observed at the medulla or cortex. In lymph nodes with positive HE staining, tumor cells were found gathered into a cluster. Additionally, some lymph nodes were disrupted by tumor cells (Figure 4).

In total, 697 lymph nodes in 45 gastric adenocarcinomas patients were examined, with a median number of 13 nodes (ranging from seven to 46) and an average number of 15. In all, lymph node micrometastasis was identified in 35 of 45 patients and in 242 of 697 nodes (MLR = 34.7%, 242/697). All these nodes showed positive CK immunohistochemical staining. Furthermore, lymph nodes micrometastasis was identified by CK immunohistochemical staining in four of 10 nodes with N_0 _determined by HE staining. Lymph node micrometastasis was also identified in 61 of 455 (13.4%) lymph nodes with negative CK immunohistochemical staining. The MLR determined by CK staining was 43.5% (303/696). Notably, the MLR determined by HE staining and CK staining showed a significant difference (*P *= 0.001) (Table 4). Whether identified by HE or CK staining, the MLR was related to lymph vessel invasion and the depth of invasion (P < 0.05) (Table 5), but was not related to gender, Lauren classification, type of histology, and blood vessel invasion.

**Table 4:** Patients with lymph node metastasis detected by HE and CK staining.

	*Lymph node metastasis* *Case No (%)*		*P*	*Lymph node metastasis* *LN No (%)*		*P*
				
	Positive	Negative		Positive	Negative	
HE	35 (77.8)	10 (22.2)	0.25	303 (43.5)	394 (56.5)	0.001
CK	39 (86.7)	6 (13.3)		242 (34.7)	455 (65.3)	

**Table 5:** Correlation between MLR grades and clinical characteristics.

*Characteristics*	*Samples*	*MLR classification (HE)*			*P*	*MLR classification (CK)*			*P*
					
		MLR_1_	MLR_2_	MLR_3_		MLR_1_	MLR_2_	MLR_3_	
Total	45	10	12	23		6	9	30	
Gender					0.607				0.508
Male	26	4	11	11		2	6	18	
Female	19	6	1	12		4	3	12	
Lauren type					0.823				0.870
Intestinal type	42	9	12	21		6	8	28	
Diffuse type	3	1	0	2		0	1	2	
Type of histology					0.808				0.833
1–2	28	5	10	13		3	7	18	
3	17	5	2	10		3	2	12	
Lymphatic vessel invasion					0.000				0.000
Negative	10	9	1	0		5	4	1	
Positive	35	1	11	23		1	5	29	
Blood vessel invasion					0.086				0.069
Negative	35	10	9	16		6	8	21	
Positive	10	0	3	7		0	1	9	
Depth of invasion					0.045				0.019
pT_1–2_	15	6	4	5		5	3	7	
pT_3–4_	30	4	8	18		1	6	23	

## Discussion

The prognosis was significantly related to pathological characteristics. MLR is a simple and effective marker that can prevent stage migration. Nonetheless, the criteria of MLR classification need to be established [9,10]. The MLR cutoff was designated as 20% (N_0_, 0%; N_1_, <20%; N_2_, >20%) in a German gastric cancer study [9,10]. Yu and colleagues designated the MLR cutoff as 25% in gastric cancer patients that underwent D2 lymphadenectomy [11]. Kodera and colleagues defined the MLR as 0%, 1% – 19%, 20% – 60% and >60% in gastric cancer patient that underwent D2 lymphadenectomy [6]. Hyung and colleagues designated 10% MLR as N_1 _stage and 25% MLR as N_2 _stage in T3 gastric cancer [5]. Additionally, the MLR was defined as ≤ 25%, ≤ 50% and >50% [4] or 0%, 1% – 10%, 11% – 25% and >25% [3]. The MLR was also classified as 0%, 0% – 30%, 30% – 50% and >50% in a Chinese study [2]. All the studies mentioned above demonstrated that the MLR is an independent prognostic factor in gastric cancer. However, more effective criteria for MLR classification need to be further elucidated.

The ROC curve has been extensively used to measure diagnostic accuracy. The ROC curve also can be used to evaluate the predictive value of the scoring system [12,13]. By using the ROC curve in the current study to determine the cutoff, the MLR proved to be an independent prognostic factor in gastric cancer. In the N_2 _stage of the JRSGC classification and N_1 _stage of the UICC classification, differences in prognosis were seen among the different MLR groups. Three-year and five-year survival rates were believed to be effective markers for gastric cancer prognosis. Therefore, the combined ROC curve with MLR is an effective strategy for drawing the curve to predict three-year and five-year survival rates.

Metastatic foci in lymph nodes, ranging from 0.2 to 2 mm, <0.2 mm, and >2 mm in diameter, were identified as lymph node micrometastasis, isolated tumor cells (ITCs), and lymph node metastasis, respectively [8]. Metastatic foci in lymph nodes were in a nonclustered or clustered distribution: a single clustered metastatic focus with a maximum diameter ranging from 0.2 to 2 mm, multiple clustered metastatic foci with the maximum sum of diameters ranging from 0.2 to 2 mm, and nonclustered metastatic foci with the maximum area size, including cancer cells, ranging from 0.2 to 2 mm [14].

Lymph node metastasis is one of the most important prognostic factors in gastric cancer. Until now, HE staining as a routine pathological examination is the good standard for the diagnosis of lymph node metastasis. However, the occurrences of lymph node micrometastasis could not be identified by routine pathological detection. Recent advances in immunohistochemical and molecular biologic techniques have made it possible to detect the lymph node micrometastasis. Cytokeratin is a component of the cytoskeleton of epithelial cells, which dose not present in the lymph nodes. Immunohistochemical examination by CK20 as one of cytokeratin family and a gene marker of tumor has been applied for longer than a decade [15] and CK20 mRNA has also successfully been detected in lymph nodes without metastasis in routine histological examination [16]. In comparison with the detection of CK20 mRNA from lymph node, the immunohistochemical examination of CK20 has some advantages such as morphological observation and utilization of retrospective investigation.

Morphologically, cancer cells in lymph nodes were described as marginal sinus, intermediate sinus, parenchymal, and diffuse types. Marginal sinus is the most common type. This may be due to migrant cancer cells that were initially arrested in the marginal sinus [14,17]. In this study, metastatic foci in lymph nodes were mainly located at the marginal sinus with a nonclustered or clustered distribution, which is consistent with metastasis theory. A previous study indicated that micrometastasis in lymph nodes had proliferating activity and had the potential for developing metastasis [18].

## Conclusion

In conclusion, our study suggests that the MLR is an independent prognostic factor in gastric cancer and, when combined with the ROC curve, is an effective strategy for drawing a curve for predicting the 3-year and 5-year survival rates. The results of lymph node micrometastasis make the MLR increase.

## Competing interests

The authors declare that they have no competing interests.

## Authors' contributions

JWY contributed in study concepts, manuscript preparation and manuscript editing. JGW carried out study design, definition of intellectual content, literature research, experimental studies, data acquisition, data analysis, statistical analysis and manuscript preparation. LHZ, BZ, XCN and BJJ contributed in clinical managements. XQL contributed in pathological studies. BJJ contributed in guarantor of integrity of the entire study, study concepts, study design and manuscript review.
